# Misidentification of a duodenal neuroendocrine tumor as an adenoma, with subsequent attempted resection by cold snare polypectomy

**DOI:** 10.1055/a-2209-9924

**Published:** 2023-12-13

**Authors:** Hirotaka Oura, Akihiro Miyakawa, Tadao Nakazawa

**Affiliations:** 1 428677 Department of Gastroenterology, Tokyo Women's Medical University Yachiyo Medical Center, Yachiyo, Japan; 2 Department of Gastroenterology, Asahi General Hospital, Asahi, Japan; 3 428677 Department of Pathology, Tokyo Women's Medical University Yachiyo Medical Center, Yachiyo, Japan

A woman in her 70s underwent esophagogastroduodenoscopy screening, which indicated a 5-mm erythematous elevated lesion on the anterior side of the duodenal bulb ( Fig. 1 ). The biopsy pathology findings indicated an adenoma, and the patient was referred to our hospital for treatment. The lesion showed minor irregularity on narrow-band imaging (NBI) ( Fig. 2 **,** Fig. 3 ). However, combined with the result of the biopsy, we diagnosed it as a low-grade adenoma. We attempted to resect it by cold snare polypectomy, but resistance during snaring made resection impossible. In addition, white mucus flowed from the tumor during snaring ( Fig. 4 **;** Video 1 ). The tumor was successfully resected en bloc by endoscopic mucosal resection. Pathology findings showed a G1 neuroendocrine tumor (NET; World Health Organization classification) with negative margins ( Fig. 5 **a** ). There was scattered submucosal retention of mucin in Brunner’s gland adjacent to the NET ( Fig. 5 **b,c** ), which was thought to have drained out under pressure during snaring. The retention of the mucin may have occurred due to obstruction by the NET or inflammation, because reactive lymphoid follicles were also seen near the NET.

**Fig. 1:**
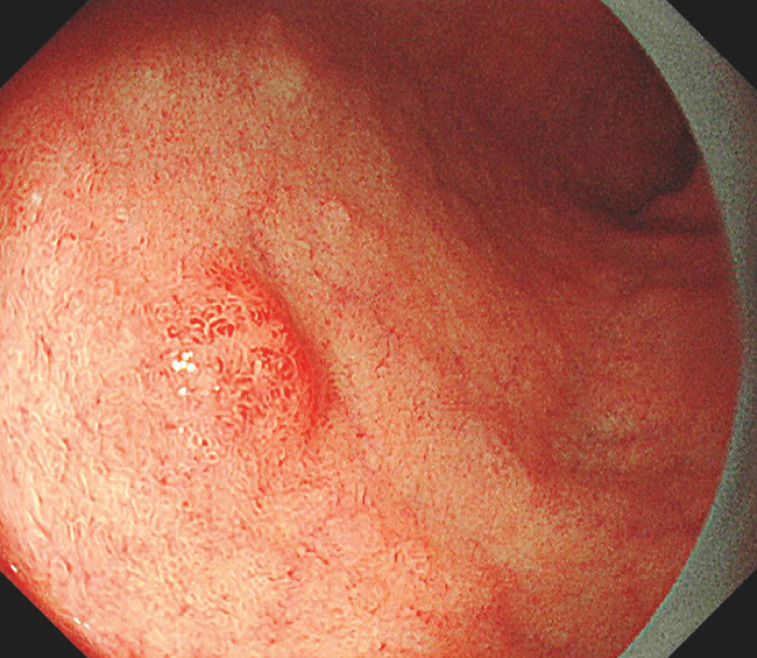
White-light image reveals an 5-mm erythematous elevated lesion on the anterior side of the duodenal bulb of a woman in her 70s.

**Fig. 2:**
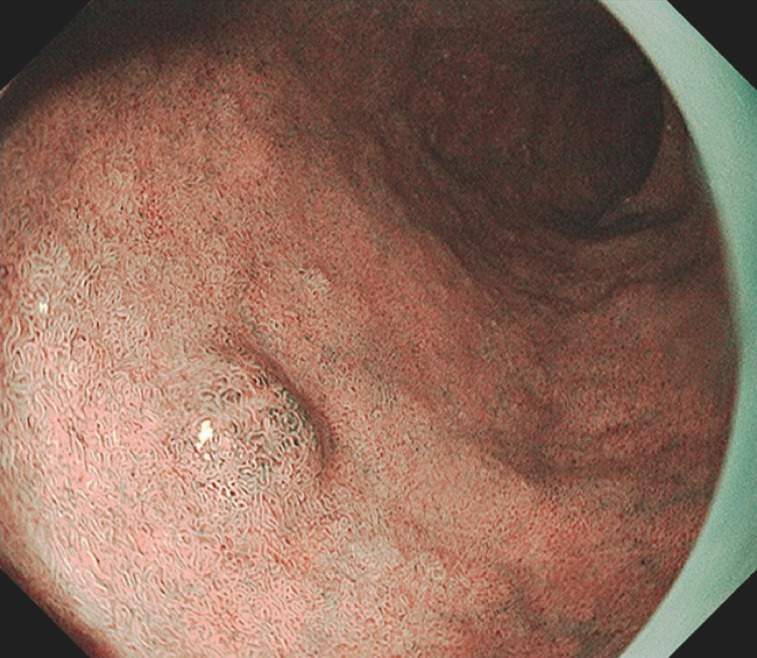
Endoscopic view of nonmagnified narrow-band imaging (NBI).

**Fig. 3:**
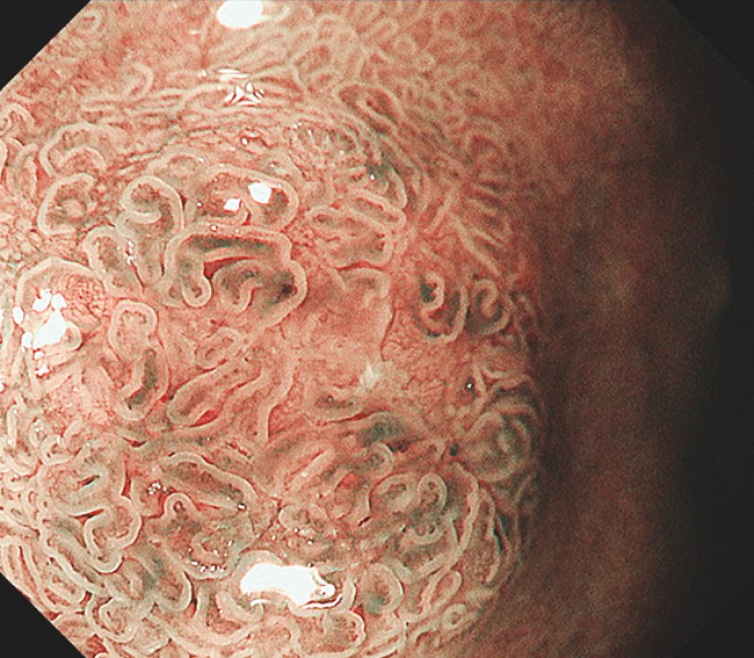
Magnified NBI image showing the lack of any white opaque substance in the lesion.

**Fig. 4:**
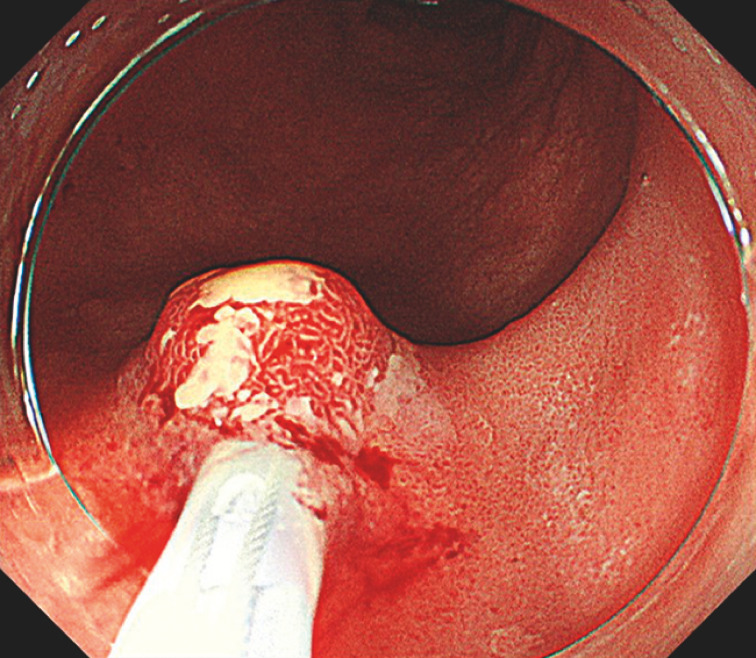
White mucus flowed from the tumor during snaring. Resection by cold snare polypectomy was not possible due to resistance during snaring.

Neuroendocrine tumor in the duodenal bulb misidentified as an adenoma, with resection attempted via cold snare polypectomy.Video 1

**Fig. 5:**
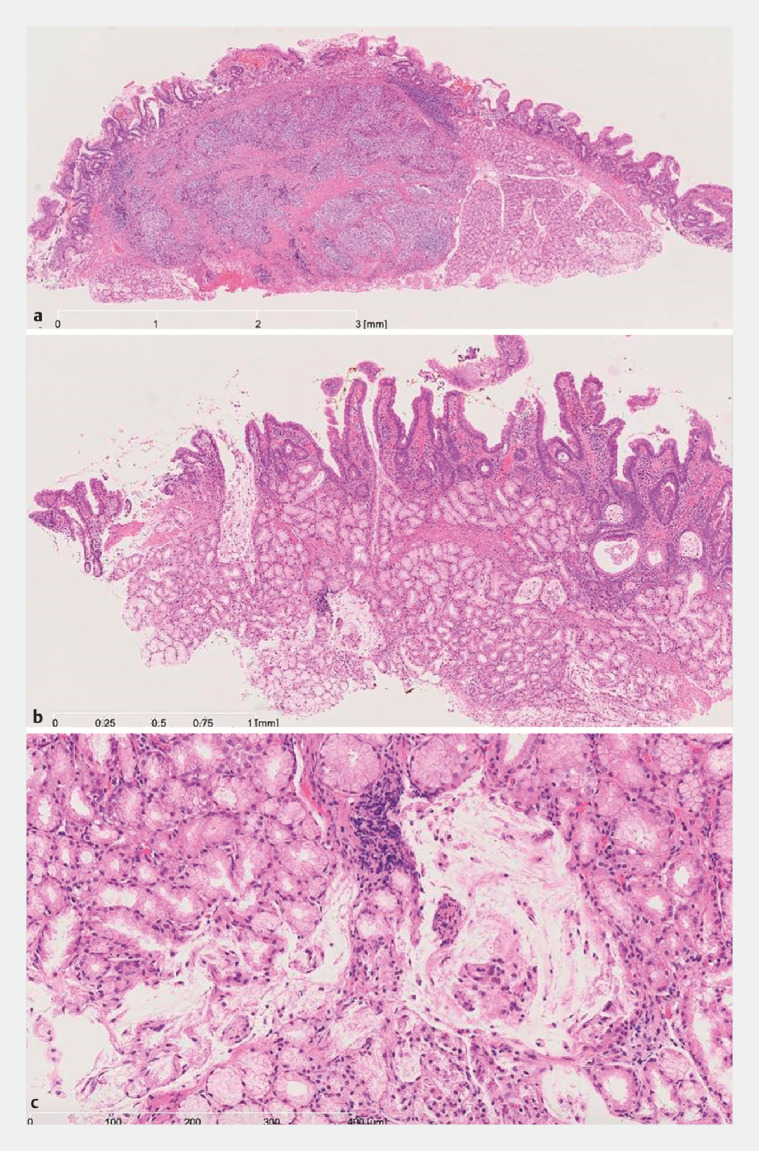
Pathology findings of the resected specimen (hematoxylin–eosin staining). **a** Loupe image of the tumor. The tumor was diagnosed as a neuroendocrine tumor (NET), G1 according to the World Health Organization classification, with negative margins. **b** Histological image of the nontumor area. There was scattered submucosal retention of mucin in Brunner’s gland adjacent to the NET. **c** Histological image around the mucus retention.

On the basis of the biopsy result, this case was diagnosed preoperatively as a low-grade adenoma. However, when reviewed retrospectively, it was noted that the lesion lacked any white opaque substance (WOS) in the magnified NBI images ( Fig. 3 ), implying that it was not a typical intestinal-type superficial duodenal epithelial tumor (SDET) 1 2 . In the duodenal bulb, the incidence of NET and the gastric type of SDET, which is considered more malignant than the intestinal type, is higher 3 4 . Treatment strategy for lesions without WOS in the duodenal bulb should be carefully considered, including endoscopic ultrasound and magnified NBI observation, because SDET biopsies tend to be inaccurate 5 . Therefore, it may be prudent to refrain from attempting cold snare polypectomy on the basis of biopsy results alone.

Endoscopy_UCTN_Code_CCL_1AB_2AZ_3AB

## Notes

Endoscopy E-Videos https://eref.thieme.de/e-videos
*E-Videos* is an open access online section of the journal *Endoscopy* , reporting on interesting cases and new techniques in gastroenterological endoscopy. All papers include a high-quality video and are published with a Creative Commons CC-BY license. Endoscopy E-Videos qualify for HINARI discounts and waivers and eligibility is automatically checked during the submission process. We grant 100% waivers to articles whose corresponding authors are based in Group A countries and 50% waivers to those who are based in Group B countries as classified by Research4Life (see: https://www.research4life.org/access/eligibility/ ). This section has its own submission website at https://mc.manuscriptcentral.com/e-videos .
